# Students’ perception of the educational environment at King Saud bin Abdulaziz University for health sciences using DREEM tool

**DOI:** 10.1186/s12909-023-05004-7

**Published:** 2024-01-08

**Authors:** Mohammed Awawdeh, Lama A. Alosail, Maram Alqahtani, Afrah Almotairi, Rahaf N. Almikhem, Rahaf A. Alahmadi, Aida A. Aldughaither, Khalid A. Abalkhail

**Affiliations:** 1 Department of Preventive Dental Science, College of Dentistry, King Saud bin Abdulaziz University for Health Sciences, P.O. Box 3660, Riyadh, 11481 Saudi Arabia; 2 King Abdullah International Medical Research Center, Ministry of National Guard-Health affairs, P.O. Box 3660, Riyadh, 11481 Saudi Arabia; 3 Department of Periodontics, King Abdulaziz Medical City, Ministry of National Guard-Health affairs, P.O. Box 3660, Riyadh, 11481 Saudi Arabia; 4 College of Dentistry, King Saud bin Abdulaziz University for Health Sciences, P.O. Box 3660, Riyadh, 11481 Saudi Arabia; 5 Department of Restorative Dentistry, King Abdulaziz Medical City, Ministry of National Guard, P.O. Box 3660, Riyadh, 11481 Saudi Arabia; 6 King Saud Medical City, Ministry of Health, Riyadh, Saudi Arabia; 7 College of Medicine, King Saud bin Abdulaziz University for Health Sciences, P.O. Box 3660, Riyadh, 11481 Saudi Arabia; 8 Family Medicine and Primary Health Care Department, Ministry of National Guard, P.O. Box 3660, Riyadh, 11481 Saudi Arabia

**Keywords:** DREEM, Educational environment, Students’ perception

## Abstract

**Background:**

Students’ learning results are influenced by the educational environment. The best learning environment is created when students are involved in the evaluation process of their education. The purpose of this study was to evaluate students’ perceptions of their learning environment at King Saud bin Abdulaziz University for Health Sciences (KSAU-HS) in Riyadh using the Dundee Ready Education Environment Measure (DREEM) instrument.

**Methods:**

This observational cross-sectional study was conducted through an online questionnaire using the Arabic version of the DREEM tool. Students from six colleges at KSAU-HS Riyadh campus were asked to complete the questionnaire through emails. The study was carried out between November 2021 and April 2022. Descriptive statistics and inferential statistics were performed for DREEM as both a continuous (two-way ANOVA test) and categorical variable (Chi-squared and Monte-Carlo test).

**Results:**

A total of 370 students completed the questionnaire. The overall DREEM score for the study was 125.88/200, with a standard deviation of 58.79. SSP items received the highest scores, while SAP items earned the lowest scores. The college and the academic level showed statistically significant differences in the DREEM overall score and the five subscales, whereas gender showed no significant difference. The college of pharmacy scored the highest total DREEM score (140.35 ± 27.75), and scored higher among the five subscales than both colleges of dentistry (114.13 ± 29.74) and medicine (113.87 ± 33.03). Students in their third year had the greatest overall DREEM score (132.23 ± 29.76), and scored higher in SPL, SPA and SSP compared to students in their sixth year, in which the total DREEM score was (111.65 ± 27.58).

**Conclusions:**

Students of KSAU-HS generally perceived the educational environment as having more positive than negative. The educational level and college differed significantly in the overall DREEM score and the five subscales. Junior students had better perception of the educational environment and they differed significantly in the SPL and SPA subscales. The faculty of pharmacy had higher scores in the overall DREEM and the five subsequent scales than colleges of dentistry and medicine. Further research is needed in order to optimize the educational environment by investigating different solutions.

## Introduction

The educational environment is the setting that students are exposed to or perceive as having a substantial influence on their learning results [1]. In recent years, the field of educational research has gained significant attention, with various research tools available to assess the educational environment such as surveys, interviews and case studies. The educational environment was found to have a significantly positive association with student’s quality of learning, and it can affect their cognitive outcomes [2]. In light of the rapid advance in educational resources and the wide variety of teaching methods available, universities are continuously seeking improvement by investigating their learning environment and student outcomes [3]. A study conducted by Tadesse et al. among 1121 undergraduate students in 3 Ethiopian universities with the aim to investigate the relationship between the learning environment and students’ learning outcomes. They found that student’s perceptions of constructivist learning environments were significantly associated with learning outcomes [4].

Students are considered keystone in the learning process, and a shift towards a student-centred learning is now increasingly being adopted. Involving students in evaluating their learning environment is one of the steps towards achieving an education of higher quality [4–6]. 28 different tools are available to assess the learning environment, 15 are used in undergraduate schools and 15 in postgraduate programs. Among them, only 4 were used in both settings which are the Dundee Ready Education Environment Measure (DREEM), the Veterans Affairs Learners’ Perceptions Survey (VA LPS), the Postgraduate Hospital Educational Environment Measure (PHEEM), and the Surgical Theatre Educational Environment Measure (STEEM). The main difference between these tools is the setting into which the questions are focused on. The tools with the highest validity reported were Pololi’s tool and graduate medical education (ACGME), although were not used in subsequent publications [7]. DREEM tool was established by Roff et al. in 1997 [8]. It is a 50-item tool divided into 5 subscales which are perceptions of learning, teaching, academic self-perception, atmosphere and social self-perception. DREEM tool is the most widely used among the learning environment tools developed, despite its low validity [7, 9]. Hammon SM et al. explored the psychometric properties of DREEM and found the overall internal consistency to be acceptable, with two subscales (Student perception of Atmosphere and Students’ social self-perception) showing low internal consistency [10]. Soemantri et al. conducted a systematic review to assess the validity and reliability of different learning environment instruments. They concluded that DREEM is the most suitable in assessing the educational environment among undergraduates and was found to be consistently reliable among different countries [11]. The DREEM tool has been used in several institutions around Europe, Africa, Asia, and America, and it has been translated into eight languages, one of which is the Arabic language. DREEM tool was utilized in different regions of Saudi Arabia in over 25 medical colleges in Jeddah, Makkah, Dammam, Qassim, Jazan and Riyadh [12–19].

In 2005, King Saud bin Abdul Aziz University for Health Sciences (KSAU-HS) was founded. The first public specialized healthcare university not only in Saudi Arabia but also in the Middle East. The main campus of KSAU-HS is in Riyadh, with other two campuses in Al-Ahsa and Jeddah. The Riyadh main campus contains seven colleges: the College of Medicine, the College of Pharmacy, the College of Dentistry, the College of Nursing, the Faculty of Public Health and Health Informatics, the College of Applied Medical Sciences, and the College of Science and Health Professions (COSHP). KSAU-HS’s mission is to deliver academic programs in an environment that promotes excellence in creative learning and scientific research.

The DREEM instrument was used to examine the educational environment in the Faculty of Nursing at KSAU-HS, Jeddah branch. The average DREEM score was 129.70/200, indicating a more positive environment with the lowest score recorded for the students’ Perceptions of the Atmosphere [16]. A recent publication by Zawawi et al. examined the educational environment in the college of medicine, KSAU-HS, Riyadh branch. The total DREEM score in their study was 110/200, indicating a more positive environment with no difference between genders or different batches [11]. This is in agreement with other local studies conducted in other universities such as Umm Al-Qura University in Makkah and Mustaqbal University in Al-Qassim, where students viewed the educational environment as positive [17, 18]. However, in Umm Al-Qura University, the perception of atmosphere received the lowest score. In contrast, the lowest score for students in Mustaqbal University was for social self-perception, and the highest was for student’s perception of learning. Previous local studies utilizing DREEM tool were mostly limited to one college. Moreover, some of the articles used the English-version, which affected students’ understanding of the items [15]. Other studies did not mention which version was used. Up to our knowledge, no research was conducted in KSAU-HS comparing all colleges. Therefore, the purpose of the present study was to evaluate the educational environment in all colleges of KSAU-HS in Riyadh using DREEM tool. The hypothesis of the present study is that gender, academic year, and college will have an impact on the perceived educational environment measured by the DREEM scores. Hence, the null hypothesis is that DREEM score is not affected by the characteristics of the sample within the same university. The findings of this study will be useful in identifying possible areas for improvement and may be utilized for future comparisons.

## Materials and methods

### Design of the study

This observational, cross-sectional study was carried out using an online questionnaire administered by Online surveys, formerly known as Bristol Online Survey (BOS) (https://www.onlinesurveys.ac.uk/). Students were recruited using emails. The research was conducted between November 2021 and April 2022. Ethical approval was obtained from King Abdullah International Medical Research Centre (KIMARC; Ref. no. IRBC/2470/21).

### Sample size

During the study period, 2602 students were enrolled in the colleges of medicine, nursing, dentistry, pharmacy, science and health professions, and applied medical sciences at KSAU-HS in Riyadh. Raosoft® software was used to compute the sample size. A minimum of 335 students was required to attain a 95% confidence level, with a margin of error of 5% and a 50% response distribution. Convenient sampling method was used to obtain 370 students, including at least 50 students from each college. The Faculty of Public Health and Health Informatics was excluded as it does not have undergraduate program.

### Measures

The participants of this study were asked to fill two sections: section A-demographical data (Table 1) and section B– The Arabic version of DREEM tool utilized by previous studies was used to insure proper understanding of each item [13].

**Table 1:** Demographical data

1. Your gender:	❑ Male ❑ Female
2. What is your current level?	❑ Preparatory years (first\second-year) ❑ Third-year ❑ Fourth-year ❑ Fifth-year ❑ Sixth-year
3. Which college are you studying in?	❑ College of Science and Health Professions ❑ College of Medicine ❑ College of Dentistry ❑ College of Pharmacy ❑ College of Nursing ❑ College of Applied Medical Sciences

DREEM tool consists of fifty items with each item scored on a five-point Likert scale with 4 = strongly agree, 3 = agree, 2 = unsure, 1 = disagree, and 0 = strongly disagree. DREEM tool measures five domains of the students’ perceptions of their institute:

- Students’ Perception of Learning (SPL): 12 items.
- Students’ Perceptions of Teachers (SPT):11 items.
- Students’ Academic self-Perceptions (SAP): 8 items.
- Students’ Perceptions of Atmosphere (SPA): 12 items.
- Students’ Social self-Perceptions (SSP): 7 items.

Mean total scores for all subscales were interpreted according to the practical guide using the DREEM written by McAleer and Roff as shown in Table 2 [8]. All reverse statements were calculated as a reverse score following the scoring guidelines.

**Table 2:** Guide for interpretation of the DREEM score

Total DREEM score	Score	Interpretation
	0–50	Very poor
	51–100	Plenty of problems
	101–150	More positive than negative
	151–200	Excellent

### Statistical analysis

All data were coded and analysed using the Statistical Package for the Social Sciences (SPSS©) program, version 25 (IBM Corp. Released 2017. IBM SPSS Statistics for Windows, Version 25.0. Armonk, NY: IBM Cor). Cronbach’s Alpha score was calculated to determine internal consistency of the tool. The scores from DREEM were presented both as continuous and categorical data (4-point Likert scale) based on the DREEM analysis guide. Continuous variables were presented as means and standard deviations, and categorical variables were presented as frequencies and percentages. Two-way (ANOVA) was used to compare the mean scores between the different academic levels and colleges. Following that, Chi-squared and Monte-Carlo test was used to compare the categorical scores of DREEM. A p-value of less than 0.05 was considered statistically significant.

## Results

The questionnaire had been completed by 370 students in total. There were 145 males and 225 females among them, or 60.8% and 39.2%, respectively. Table 3 displays the response rate for each academic year among various colleges.

**Table 3:** Characteristics of students who participated in the study including gender, academic level, and college (N = 370)

Student		Count	Column N %
Your gender:	Female	225	60.8%
	Male	145	39.2%
What is your current level?	Preparatory years (first\second-year)	114	30.8%
	Third-year	70	18.9%
	Fourth-year	106	28.6%
	Fifth-year	54	14.6%
	Sixth-year	26	7.0%
Which college are you studying in?	College of Applied Medical Sciences	64	17.3%
	College of Nursing	66	17.8%
	College of Pharmacy	69	18.6%
	College of Dentistry	52	14.1%
	College of Medicine	61	16.5%
	COSHP	58	15.7%

*Note*: COSHP stands for college of science and health professions

The Cronbach’s alpha coefficient for the total DREEM score value is 0.953, which indicates high internal reliability of our sample. The lowest scores were detected in SSP (0.718) and SPL (0.781), which are still good according to the general rule of interpretation. (Table 4)

**Table 4:** Cronbach Alpha scores for the total DREEM scale and subscales

Subscale	Number of questions	Cronbach’s alpha
SPL: Students’ perception of learning	12	0.781
SPT: Students’ perception of teachers	11	0.840
SAP: Students’ academic self-perception	8	0.852
SPA: Students’ perception of atmosphere	12	0.857
SSP: Students’ social self-perception	7	0.718
Total DREEM Score	50	0.953

The study’s total DREEM score was 125.88/200 with a 58.79 standard deviation. This result shows that more students think favourably of the learning environment at KSAU-HS. Table 5 displays the results of the sample’s DREEM subscale scores. The means of most of the subscales were greater than 50%. The SSP items received the highest scores (mean = 3.246), while the SPA items received the lowest scores (mean = 1.068).

**Table 5:** The DREEM domains with total and individual scores

Domain	Interpretation	Count	Percentage
**SPL**: Students’ perception of learning: 12 items	Very poor	14	3.8%
	Teaching is viewed negatively	82	22.2%
	A more positive approach	210	56.8%
	Teaching highly thought of	64	17.3%
**SPT**: Students’ perception of teachers: 11 items	Abysmal	11	3.0%
	In need of some retraining	62	16.8%
	Moving in the right direction	182	49.2%
	Model teachers	115	31.1%
**SAP**: Students’ academic self-perception: 8 items	Feeling of total failure	18	4.9%
	Many negative aspects	96	25.9%
	Feeling more on the positive side	189	51.1%
	Confident	67	18.1%
**SPA**: Students’ perception of atmosphere: 12 items	A terrible environment	14	3.8%
	There are many issues that need to be changed	69	18.6%
	A more positive atmosphere	179	48.4%
	A good feeling overall	108	29.2%
**SSP**: Students’ social self-perception: 7 items	Miserable	1	0.3%
	Not a nice place	74	20.0%
	Not very bad	240	64.9%
	Very good socially	55	14.9%
**Total DREEM** Score 50 Items (200)	Very poor	10	2.7%
	Plenty of problems	58	15.7%
	More positive than negative	212	57.3%
	Excellent	90	24.3%

### Students’ perceptions and their academic level

**Fig. 1:**
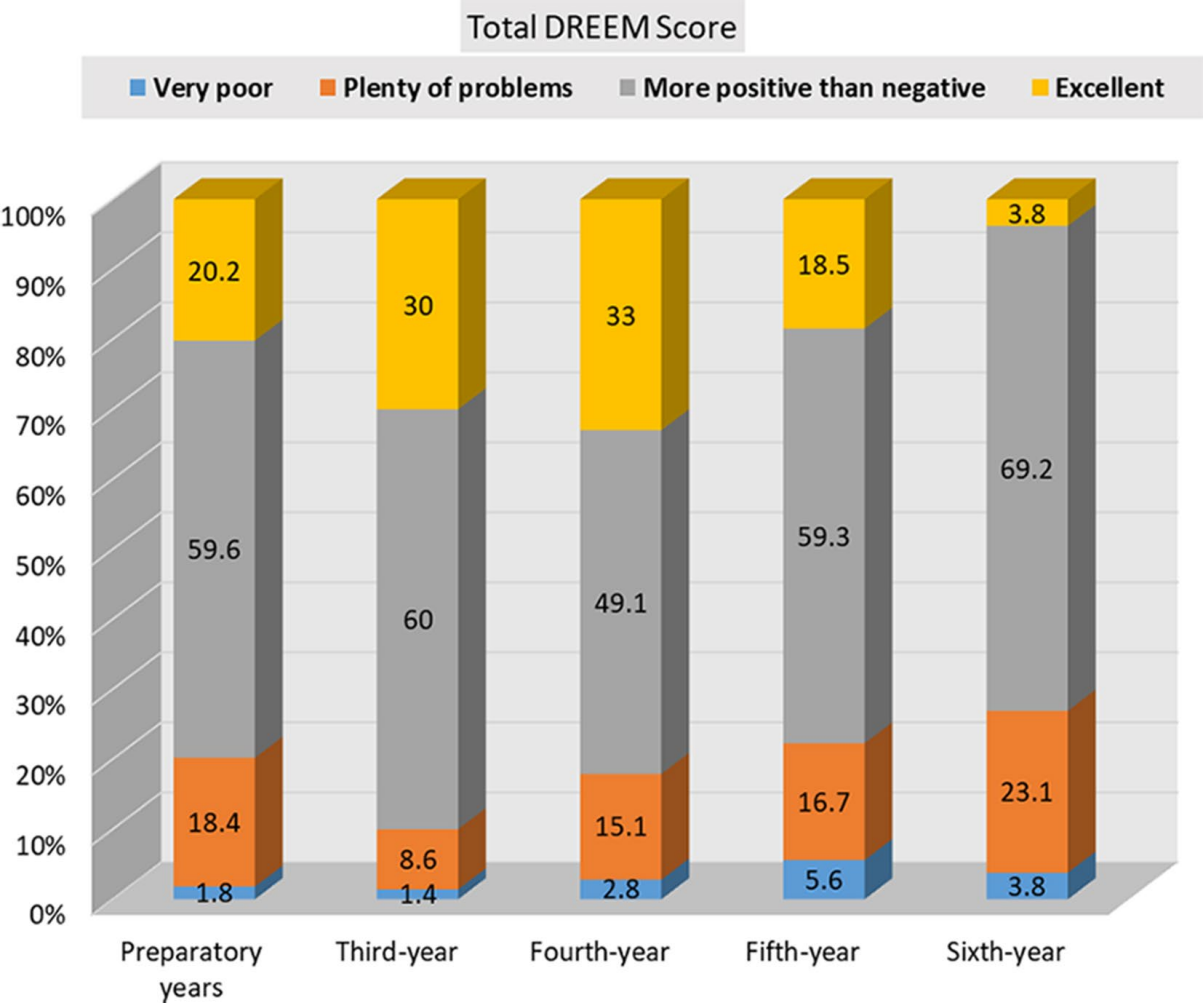
DREEM total score frequency by academic level

The DREEM overall scale revealed statistically significant difference between the fourth-year students (130.34 ± 34.23) compared to the sixth-year students (111.65 ± 27.58). The SPL, SPA, SSP subscales showed significant differences between junior and senior students, with P = 0.002, 0.013, and 0.021 respectively (Table 6). DREEM subscale categories analysed by academic level is presented in Table 7; Fig. 1.

Two-way ANOVA analysis for the academic level effect on the overall DREEM score shows the following pairs to be statistically significant; third-year × sixth-year (P value = 0. 027), fourth-year × sixth-year (P value = 0. 041). For the analysis of subscales effect on the academic level, the following pairs were statistically significant in relation to SPL, preparatory years (first\second-year) × sixth-year (P value = 0.024), third-year × sixth-year (P value = 0 0.002), fourth-year × sixth-year (P value = 0 0.010). For SPA, third-year ×sixth-year (P value = 0. 039), fourth-year × sixth-year (P value = 0.040). For SSP, third-year * sixth-year (P value = 0.061), fourth-year * sixth-year (P value = 0.031).

**Table 6:** Mean scores for DREEM domains for the overall sample and the significant differences between the academic levels

	Preparatory years (first\second-year)	Third-year	Fourth-year	Fifth-year	Sixth-year		
Domain	Mean ± SD					P value	Interpretation*
**SPL**: Students’ perception of learning	29.35 (7.47)	30.83 (7.58)	29.82 (8.47)	26.76 (9.19)	24.38 (7.27)	**0.002**	A more positive approach
**SPT: Students’ perception of teachers**	28.07 (8.02)	28.67 (8.33)	30.17 (8.35)	27.87 (8.56)	26.92 (8.40)	0.099	Moving in the right direction
**SAP: Students’ academic self-perception**	19.29 (5.61)	19.10 (5.61)	19.55 (6.04)	18.09 (5.75)	17.46 (4.36)	0.111	Feeling more on the positive side
**SPA: Students’ perception of atmosphere**	31.17 (9.02)	30.71 (9.83)	32.34 (10.06)	28.78 (10.11)	26.81 (7.69)	**0.013**	A more positive atmosphere
**SSP: Students’ social self-perception**	18.06 (4.16)	17.67 (3.63)	18.46 (4.24)	17.28 (3.58)	16.08 (3.70)	**0.021**	Not very bad
**Total DREEM Score 50 Items (200)**	127.20 (31.70)	125.04 (33.00)	130.34 (34.23)	118.78 (34.36)	111.65 (27.58)	**0.014**	More positive than negative

*Note*: P value < 0.05 considered significant, ^*^Interpretation guide adapted from McAleer (2001)

**Table 7:** DREEM subscale categories analyzed by academic level

Interpretation	Preparatory years (first\second-year) N = 114(%)	Third-year N = 70(%)	Fourth-year N = 106(%)	Fifth-year N = 54(%)	Sixth-year N = 26(%)	Test of significance
**Students’ perception of learning**						
Very poor	2(1.8)	1(1.4)	4(3.8)	5(9.3)	2(7.7)	χ^2MC^=28.39 P = 0.005*
Teaching is viewed negatively	24(21.1)	8(11.4)	23(21.7)	17(31.5)	10(38.5)	
A more positive approach	72(63.2)	46(65.7)	55(51.9)	23(42.6)	14(53.8)	
Teaching highly thought of	16(14.0)	15(21.4)	24(22.6)	9(16.7)	0	
**Students’ perception of teachers**						
Abysmal	2(1.8)	1(1.4)	3(2.8)	3(5.6)	2(7.7)	χ^2MC^=15.79 P = 0.201
In need of some retraining	26(22.8)	8(11.4)	15(14.2)	9(16.7)	4(15.4)	
Moving in the right direction	53(46.5)	38(54.3)	46(43.4)	30(55.6)	15(57.7)	
Model teachers	33(28.9)	23(32.9)	42(39.6)	12(22.2)	5(19.2)	
**Students’ academic self-perception**						
Feeling of total failure	5(4.4)	2(2.9)	6(5.7)	4(7.4)	1(3.8)	χ^2MC^=15.79 P = 0.268
Many negative aspects	30(26.3)	14(20.0)	25(23.6)	16(29.6)	11(42.3)	
Feeling more on the positive side	62(54.4)	37(52.9)	25(46.3)	25(46.3)	14(53.8)	
Confident	17(14.9)	17(24.3)	9(16.7)	9(16.7)	0	
**Students’ perception of atmosphere**						
A terrible environment	5(4.4)	1(1.4)	3(2.8)	3(5.6)	2(7.7)	χ^2MC^=14.54 P = 0.286
There are many issues that need to be changed	20(17.5)	11(15.7)	18(17.0)	13(24.1)	7(26.9)	
A more positive atmosphere	59(51.8)	36(51.4)	44(41.5)	26(48.1)	14(53.8)	
A good feeling overall	30(26.3)	22(31.4)	41(38.7)	12(22.2)	3(11.5)	
**Students’ social self-perception**						
Miserable	0	0	0	0	1(3.8)	χ^2MC^=43.25 P < 0.001*
Not a nice place	22(19.3)	5(7.1)	23(21.7)	14(25.9)	10(38.5)	
Not very bad	81(71.1)	56(80.0)	56(52.8)	33(61.1)	14(53.8)	
Very good socially	11(9.6)	9(12.9)	27(25.5)	7(13.0)	1(3.8)	
**Total DREEM Score**						
Very poor	2(1.8)	1(1.4)	3(2.8)	3(5.6)	1(3.8)	χ^2MC^=18.72 P = 0.095
Plenty of problems	21(18.4)	6(8.6)	16(15.1)	9(16.7)	6(23.1)	
More positive than negative	68(59.6)	42(60.0)	52(49.1)	32(59.3)	18(69.2)	
Excellent	23(20.2)	21(30.0)	35(33.0)	10(18.5)	1(3.8)	

*Note*: MC: Monte Carlo test, * P value < 0.05 considered significant

### Students’ perceptions and their college

**Fig. 2:**
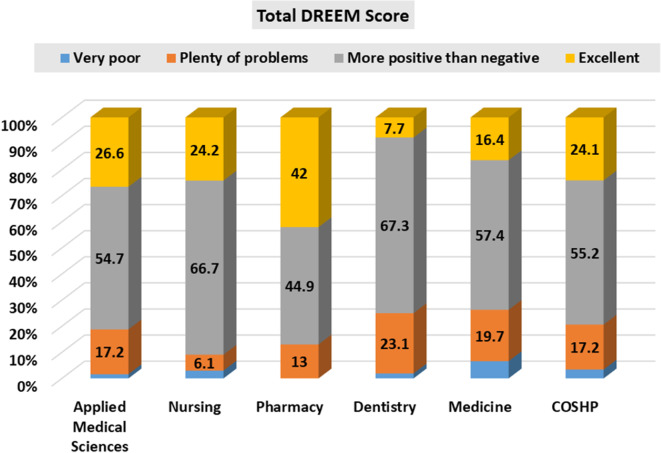
DREEM total score frequency by college

Table 8 compares the mean score of DREEM domains across colleges. Statistically, there was a significant difference in the overall DREEM score of the college of pharmacy (140.35 ± 27.75) compared to both colleges of dentistry (114.13 ± 29.74) and colleges of medicine (113.87 ± 33.03); p < 001. DREEM subscale categories analysed by college are presented in Table 9; Fig. 2. Two-way ANOVA for the college effect on the overall DREEM score shows the following pairs to be statistically significant; college of pharmacy × college of dentistry (P value = < 0.001), college of pharmacy × college of medicine (P value = < 0.001).

Comparisons of DREEM subscale categories by college is presented in Table 9. For the analysis of subscales effect on the college, the following pairs were statistically significant in relation to SPL SPT, ASP, SPA and SSP. For SPL, college of nursing × college of dentistry (P value = 0 0.017), college of nursing × college of medicine (P value = 0 0.015), college of pharmacy × college of dentistry (P value = < 0 0.001), college of pharmacy ×college of medicine (P value = < 0 0.001). For SPT, college of pharmacy * college of dentistry (P value = 0. 007), college of pharmacy * college of medicine (P value = < 0 0.001). For ASP, college of nursing × college of dentistry (P value = 0. 016), college of nursing × college of medicine (P value = 0.023), college of pharmacy × college of dentistry (P value = < 0 0.001), college of pharmacy × college of medicine (P value = < 0 0.001). for SPA, college of pharmacy × college of dentistry (P value = < 0 0.001), college of pharmacy × college of medicine (P value = < 0 0.001). for SSP, college of pharmacy × college of dentistry (P value = 0.013) (See Table 9).

**Table 8:** Mean scores for DREEM domains for the overall sample and the significant differences between the different colleges

Domain	COAMS^1^ M±(SD)	CON^2^ M±(SD)	COP^3^ M±(SD)	COD^4^ M±(SD)	COM^5^ M±(SD)	COSHP^6^ M±(SD)	P	Interpretation**
**SPL**: Students’ perception of learning	29.34 (8.17)	30.06 (7.75)	32.97 (6.68)	25.52 (8.05)	25.64 (8.26)	29.59 (8.07)	*<0.001	A more positive approach
SPT: Students’ perception of teachers	29.09 (8.17)	29.71 (8.08)	32.33 (7.38)	27.35 (7.78)	26.20 (8.36)	28.29 (8.15)	*<0.001	Moving in the right direction
**SAP**: Students’ academic self-perception	19.42 (5.42)	20.14 (5.29)	21.45 (4.81)	16.96 (4.97)	17.20 (6.08)	19.17 (5.84)	*<0.001	Feeling more on the positive side
**SPA**: Students’ perception of atmosphere	30.78 (9.23)	32.00 (9.43)	34.87 (8.56)	27.87 (8.32)	27.48 (9.56)	31.31 (10.13)	*<0.001	A more positive atmosphere
**SSP**: Students’ social self-perception	18.22 (3.67)	17.98 (3.38)	18.72 (3.53)	16.44 (3.81)	17.36 (4.18)	17.84 (4.28)	0.031	Not very bad
**Total DREEM** Score 50 Items (200)	126.86 (31.89)	129.89(31.16)	140.35 (27.75)	114.13 (29.74)	113.87 (33.03)	126.21 (34.39)	*<0.001	More positive than negative

*Note*: * P value < 0.05 considered significant, **Interpretation guide adapted from McAleer (2001)

1: College of applied medical sciences, 2: College of nursing, 3: College of pharmacy, 4: College of Dentistry, 5: College of medicine and 6: College of Science and Health Professions. COSHP

**Table 9:** DREEM subscale categories analyzed by college

Interpretation	College of Applied Medical Sciences N = 64(%)	College of Nursing N = 66(%)	College of Pharmacy N = 69(%)	College of Dentistry N = 52 (%)	College of Medicine N = 61(%)	COSHP N = 58(%)	Test of significance
**Students’ perception of learning**							
Very poor	3(4.7)	1(1.5)	0	3(5.8)	6(9.8)	1(1.7)	χ^2MC^=42.19 P < 0.001*
Teaching is viewed negatively	14(21.9)	11(16.7)	6(8.7)	19(36.5)	19(31.1)	13(22.4)	
A more positive approach	36(56.2)	42(63.6)	40(58.0)	25(48.1)	32(52.5)	35(60.3)	
Teaching highly thought of	11(17.2)	12(18.2)	23(33.3)	5(9.6)	4(6.6)	9(15.5)	
**Students’ perception of teachers**							
Abysmal	1(1.6)	2(3.0)	0	2(3.8)	4(6.6)	2(3.4)	χ^2MC^=21.02 P = 0.136
In need of some retraining	13(20.3)	8(12.1)	8(11.6)	10(19.2)	13(21.3)	10(17.2)	
Moving in the right direction	29(45.3)	33(50.0)	29(42.0)	30(57.7)	32(52.5)	29(50.0)	
Model teachers	21(32.8)	23(34.8)	32(46.4)	10(19.2)	12(19.7)	17(29.3)	
**Students’ academic self-perception**							
Feeling of total failure	2(3.1)	4(6.1)	0	3(5.8)	6(9.8)	3(5.2)	χ^2MC^=39.47 P = 0.001*
Many negative aspects	16(25.0)	9(13.6)	10(14.5)	21(40.4)	24(39.3)	16(27.6)	
Feeling more on the positive side	35(54.7)	40(60.6)	38(55.1)	26(50.0)	22(36.1)	28(48.3)	
Confident	11(17.2)	13(19.7)	21(30.4)	2(3.8)	9(14.8)	11(19.0)	
**Students’ perception of atmosphere**							
A terrible environment	1(1.6)	3(4.5)	0	2(3.8)	5(8.2)	3(5.2)	χ^2MC^=37.11 P = 0.001*
There are many issues that need to be changed	14(21.9)	6(9.1)	10(14.5)	15(28.8)	15(24.6)	9(15.5)	
A more positive atmosphere	32(50.0)	36(54.5)	25(36.2)	29(55.8)	30(49.2)	27(46.6)	
A good feeling overall	17(26.6)	21(31.8)	34(49.3)	6(11.5)	11(18.0)	19(32.8)	
**Students’ social self-perception**							
Miserable	0	0	0	0	1(1.6)	0	χ^2MC^=43.25 P = 0.144
Not a nice place	11(17.2)	9(13.6)	9(13.0)	19(36.5)	15(24.6)	11(19.0)	
Not very bad	41(64.1)	48(72.7)	47(68.1)	27(51.9)	37(60.7)	40(69.0)	
Very good socially	12(18.8)	9(13.6)	13(18.8)	6(11.5)	8(13.1)	7(12.1)	
**Total DREEM Score**							
Very poor	1(1.6)	2(3.0)	0	1(1.9)	4(6.6)	2(3.4)	χ^2MC^=33.01 P = 0.005*
Plenty of problems	11(17.2)	4(6.1)	9(13.0)	12(23.1)	12(19.7)	10(17.2)	
More positive than negative	35(54.7)	44(66.7)	31(44.9)	35(67.3)	35(57.4)	32(55.2)	
Excellent	17(26.6)	16(24.2)	29(42.0)	4(7.7)	10(16.4)	14(24.1)	

*Note*: * P value < 0.05 considered significant

### Students’ perceptions and their gender

The gender variable on the other hand revealed no statistically significant difference between the overall mean DREEM scores of the male students (127.2 ± 31.7) and the female students (125.04 ± 33.002); p = 0.532. DREEM subscale categories analysed by gender revealed SSP to be statistically significant (P = 0.026), where females had better social self-perception than males.

## Discussion

The KSAU-HS strategic goals are designed with a focus on developing academic programs enhancing excellence in health sciences research, creating vibrant on-campus quality of life, and developing sustainable community partnerships. Improving the educational environment of the institute is crucial for student academic achievement. With a supportive educational environment, the students would enhance their academic performance as well as their subjective learning outcomes to a higher level of learning.

The Cronbach’s alpha score of the present study was 0.953, suggesting high internal consistency, and this is consistent with other studies done in KSAU-HS and King Abdulaziz University Faculty of Dentistry (KAUFD), with scores of 0.94 and 0.93 respectively [12, 15].

The mean DREEM score of the present study was 125.88/200. Nationally, this value is higher than previous studies conducted in medical and dental schools: Jazan medical school (104.9\200), KSAU-HS (110\200), and Imam Abdulrahman bin Faisal University (112.38\200), but comparable with KAUFD (125\200) and Mustaqbal University (130.87\200) [12, 13, 15, 18, 20]. Internationally, our overall DREEM score was higher than that achieved by Cadi Ayyad university in Morocco (86.5\200), and comparable to scores obtained across 11 dental schools in Korea. (125.03\200) [21, 22].

In this study, 60.8% of respondents were females, which might be ascribed to the ease of access to female students, particularly in the college of nursing, which is only dedicated for females at KSAU-HS. No significant difference was found between genders in the overall DREEM score, which is consistent with the the study done in COM-KSAU-HS. [15] In Mustaqbal university, males had higher overall DREEM scores and even scored higher in all subscales [18]. In contrast to the study done in KAUFD in Jeddah, females scored slightly higher than male students in the mean DREEM score [12]. In our study, females had better social self-perception than males (P = 0.026). In contrast to the study done in Jazan, females across different academic levels scored lower in SSP than males [20]. Despite the fact that KSAU-HS students follow identical curricula, educational requirements, and teaching methods, they have separate campuses, which meet with the cultural and social norms followed in Saudi Arabia. This may account for the lack of significant difference between genders in the overall score.

Our study found a statistically significant variation in the total DREEM scores and the five subscales among colleges. Conversely, DREEM overall score in a study conducted at King Saud University showed no statistically significant difference among the variables investigated [23]. Up to our knowledge, the present study is the first in Saudi Arabia to assess the educational environment among different colleges in one institution, which could be attributed to the lack of differences among the variables in previous studies, as students were in the same speciality. In our study, the faculty of pharmacy had the greatest overall DREEM score, while the colleges of dentistry and medicine received the lowest total DREEM ratings. This might be related to the fact that pharmacy students have fewer clinical requirements and no competency examinations. In contrast to a study done in Damascus pharmacy school in Syria, the overall DREEM score was 89.8/200, which indicates that there are plenty of problems [24]. Our study showed that colleges of nursing and pharmacy had better perception of the educational environment among all the five subscales compared to colleges of dentistry and medicine. In a recent systematic review comparing DREEM scores among medical and dental colleges in Saudi Arabia, the overall DREEM scores ranged between 51 and 100 and 101–150, with slightly higher score in medical colleges. SPT and SPA domines were slightly higher among medical colleges than the dental scores [25].

This study showed that third-year students showed the highest total DREEM score compared to other levels, whereas sixth-year students showed the lowest total DREEM score. In relation to SPL and SPA, third year × sixth-year were found to be statically significant. Similarly, the highest DREEM score was achieved by third-year students in Dammam, and there was a significant mean difference between preclinical and clinical students in SPL and SPA domains [13]. One of the explanations mentioned was the recency of their enrolment, which possibly enhanced their experience [13]. Moreover, in a study done in King Saud University, second-year students obtained a significantly higher score (118.36 ± 15.8) compared to the interns (105 ± 21.3) [23].

One thing that could be determined with high certainty is that colleges with clinical requirements in the last years have placed an additional strain on their students, which is reflected in the stress levels of students in the last academic level. This result would emphasize the importance of recognizing course activities associated with stress and the need to support educational growth by offering healthy extracurricular activities, especially because previous research has revealed that stress levels among Saudi students are greater than the national norm [26]. It appears that colleges with clinical requirements are adding extra burden on their students, which is reflected negatively on the students’ perception of the educational environment. Also, it seems that students’ perception varies according to the year of study. Students during first and last years have lower perception of the educational environment compared with other years. In other words, the perceptions start low then increase and end low again. This might be due to the fact that students are stepping from the known to the unknown territory, as the first example demonstrates clearly when the students leave their comfort zone at the school and start their expedition into the university stage. This phenomenon reoccurs in the last year at their college as they are about to finish their study and step into the concealed career path. This variation in the student’s perceptions based on their college and stage of study seems to be a natural consequence of the discrepancies in their academic load and the different future postgraduate options offered to them.

There is a need for a sound support system, student counselling, stress management programs, and distribution of the courses evenly based on their complexity to help improve the educational environment. Using technology and artificial intelligence in education would undoubtedly be beneficial, as it has been claimed in one study that using technology and virtual reality has a positive outcome in improving knowledge retention and learning experiences [27]. Unifying the curriculum and program learning goals at the national level would also improve the educational experience, as a recent comparative assessment of clinical requirements among dentistry students in Saudi Arabia in Oral surgery revealed considerable disparities between different institutions [28].

The current study has few limitations. Firstly, students who participated in this study are from one institution, with a convenient sampling method, which limits the generalizability of the findings to other medical institutions. Secondly, the questionnaire is pre-validated with fixed choices; it may not incorporate all educational aspects relevant to KSAU-HS. Finally, due to the self-reported nature of the study, limitation might have been introduced in the results.

## Conclusion

In conclusion, students of KSAU-HS generally perceived the educational environment as having more positive than negative. The educational level and college differed significantly in the overall DREEM score and the five subscales. Junior students had better perception of the educational environment and they differed significantly in the SPL and SPA subscales. The faculty of pharmacy had higher scores in the overall DREEM and the five subscales than colleges of dentistry and medicine. Additional research is needed in these two colleges in order to optimize the educational environment by investigating different solutions. We recommend repeating this study in the same institution after implementing quality assurance tools to address the deficiency identified by this research. This study provided a comprehensive insight of the educational environment in all colleges of KSAU-HS, Riyadh campus, and further research in all branches of the university is needed for future comparisons.

## Data Availability

The datasets used and analysed during the current study are available from the corresponding author on reasonable request.

## Abbreviations

ANOVA: Analysis of variance
BOS: Bristol Online Survey
COSHP: College of Science and Health Professions
DREEM: Dundee Ready Education Environment Measure
KIMARC: King Abdullah International Medical Research Centre
KSAU-HS: King Saud bin Abdulaziz University for Health Sciences
SAP: Students’ Academic self-Perceptions
SPA: Students’ Perceptions of Atmosphere
SPL: Students’ Perception of Learning
SPT: Students’ Perceptions of Teachers
SSP: Students’ Social self-Perceptions
